# Early Diagnosis and Treatment of Mandibular Second Premolar Impaction: A Case Report

**DOI:** 10.3390/diagnostics14151610

**Published:** 2024-07-26

**Authors:** Anna-Maria Janosy, Abel Emanuel Moca, Raluca Iulia Juncar

**Affiliations:** 1 Private Dental Practice CMI Dr. Janosy Anna-Maria, 23 Corneliu Coposu Street, 410445 Oradea, Romania; ajanosy@yahoo.com; 2 Department of Dentistry, Faculty of Medicine and Pharmacy, University of Oradea, 10 Piața 1 Decembrie Street, 410073 Oradea, Romania; ralucajuncar@yahoo.ro

**Keywords:** tooth impaction, premolar, early diagnosis, treatment, case report

## Abstract

Odontogenesis, the process of tooth formation, is complex and susceptible to disruptions that can result in dental anomalies such as tooth impaction. The mandibular second premolar, though less commonly impacted than wisdom teeth, presents a unique challenge in pediatric dentistry due to its intricate etiology and the need for timely intervention. This case report aims to highlight the significance of early diagnosis and conservative management strategies in treating mandibular second premolar impaction. The case involves a pediatric patient with impacted mandibular second premolars. Initial treatment included the use of a lower removable appliance with an expansion screw to alleviate crowding, followed by a fixed space maintainer and a Haas rapid palatal expander. These interventions created the necessary space for the premolars to erupt. Self-ligating brackets were later applied, reducing friction and improving periodontal health. The patient underwent two CBCT examinations to monitor progress, which confirmed the successful eruption and alignment of the impacted premolars without the need for surgical exposure. This case underscores the effectiveness of early diagnosis and minimally invasive treatment in managing mandibular second premolar impaction. The tailored approach facilitated the natural eruption of the teeth, highlighting the importance of individualized treatment plans. Future research should focus on optimizing these conservative strategies to enhance patient outcomes in similar cases.

## 1. Introduction

Odontogenesis, or the process of tooth formation and development, is a complex and still incompletely understood process [1], which occurs in several stages [2]. Dental eruption is considered by some authors to be the final stage of odontogenesis, and it is defined as the physiological process in which the tooth moves vertically from its initial non-functional, intraosseous position to its intraoral, functional position, emerging through the bone of the alveolar process and the oral epithelium to eventually occlude with the opposing tooth [3]. This process is influenced by a variety of general factors such as genetics, nutrition, birth, socio-economic status, height, weight, craniofacial morphology, hormonal factors, and various systemic diseases [4]. It is also affected by local factors such as gingival hyperplasia, supernumerary teeth, odontomas, dental retention, and arch-length deficiencies [4]. Under the influence of these factors, the dental eruption process can experience various pathological changes [5], the early diagnosis of which is necessary to enable optimal therapeutic management of the patient and the establishment of an appropriate therapeutic plan [6].

Dental anomalies, which can result from disruptions in the processes of tooth formation, development, and eruption, can affect the shape and structure of teeth, the number of teeth, as well as the position of teeth [7]. Among positional anomalies, tooth impaction is relatively common, with a prevalence ranging from 0.8% to 3.6% in the general population [8]. Tooth impaction can affect any tooth, but it is most frequently observed with wisdom teeth [9,10]. Less frequently affected by dental impaction are incisors, canines, second premolars, and first and second molars [10]. The prevalence of impaction of mandibular second premolars generally varies between 0.2% and 0.3%, making it the third most common type of dental impaction after third molar and canine impaction [11].

The etiology of the impaction of the mandibular second premolar is complex. For instance, the lack of adequate space in the dental arch can impede the eruption of the second premolar. Even when adequate space is available, impaction can be associated with various general factors (e.g., syndromes, genetic factors, and hormonal factors) or local factors (e.g., uneven resorption of the roots of the second temporary molars, ankylosis of the second temporary molars, ectopic development of the tooth bud, and local pathologies) [11,12,13,14]. Ankylosis of the deciduous molar serves as a reliable clinical indicator for suspecting the impaction of the succeeding premolar [15]. Clinically, the second deciduous molars maintain a stable occlusal level, while the adjacent teeth erupt due to the vertical growth of the alveolar bone. Additionally, ankylosed teeth produce a distinct, sharp sound during percussion testing, in contrast to normal teeth, which emit a cushioned sound [16]. The diagnosis can be further corroborated during extraction, as ankylosed teeth are technically challenging to remove and are prone to root fractures [17]. Radiographic examination typically reveals obliteration of the periodontal ligament and increased radiopacity of the root [17]. Early diagnosis and the prompt establishment of an appropriate therapeutic plan—encompassing observation, intervention, relocation, and extraction—are essential for the normal development of the dentition [18,19].

Therapeutic intervention must be tailored to each case, as no universally valid solution exists. Orthodontic traction and repositioning can be challenging due to technical difficulties, and conservative management with surgical exposure is often unpredictable; however, both are valid treatment options [20]. Extraction of the deciduous second molar followed by the spontaneous eruption of the permanent second premolar is recommended when the inclination of the premolar is slight [20]. This minimally invasive approach is preferable during the period when the tooth still retains the potential to erupt [21].

In this context, this case report aims to underscore the importance of early diagnosis and intervention for the impaction of mandibular second premolars in a pediatric patient, emphasizing the use of a minimally invasive treatment approach.

## 2. Case Report

### 2.1. Examination, Diagnostics, and Initial Therapeutic Management

An 8-year-old boy was referred for orthodontic consultation and treatment by his general practitioner. His chief complaint was lower incisor crowding. The patient’s general and dental family history, as well as their personal medical history, revealed no significant general illnesses nor any family cases of the impaction of the second premolar.

Infraocclusion of the lower right second deciduous molar (tooth 85) and a deviation of the lower midline to the right were observed. The lower right first permanent molar (tooth 46) was mesially inclined, and the space for the eruption of the lower right second premolar (tooth 45) was reduced. The numbering of teeth was performed using the system proposed by the World Dental Federation (FDI).

**Figure 1:**
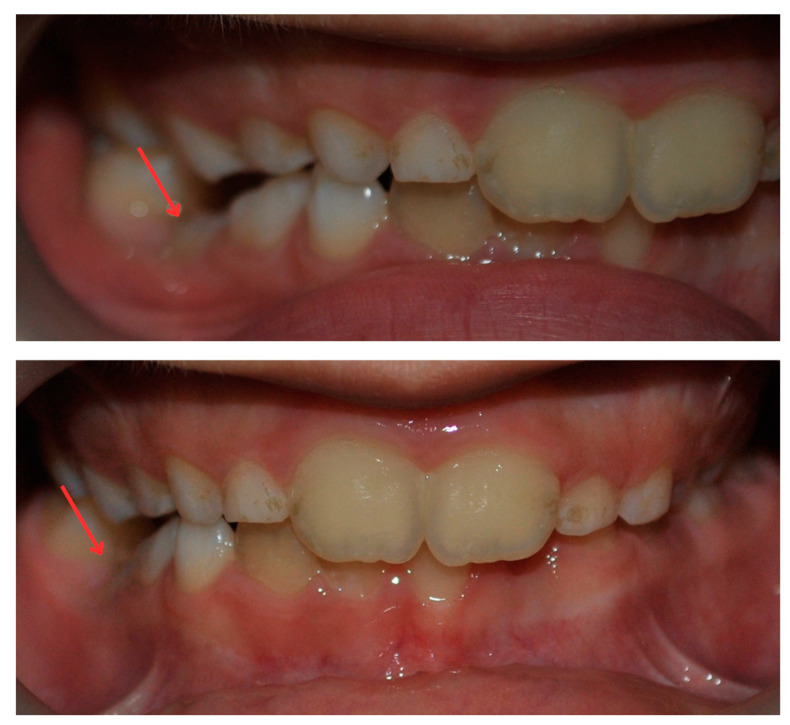
Pretreatment intraoral photographs showing the infraoccluded tooth 85.

As part of the complementary examinations, the patient underwent a panoramic radiograph as per the recommendation of the general practitioner. Additionally, intraoral photographs were taken, and an alginate impression was made to create a study cast (Figure 1). However, regrettably, the initial panoramic radiograph and study cast were lost and could not be retrieved.

Tooth 85 was found to be ankylosed, which may have been the cause of the almost horizontal position and distal inclination of tooth 45. Clinically, ankylosis was indicated by the infraocclusion of the deciduous molar (tooth 85) and the inclination of the adjacent teeth. The diagnosis was reinforced by the panoramic radiograph, which showed obliteration of the periodontal ligament and more radiopaque roots. From a technical perspective, the challenging extraction further supported this diagnosis.

As an initial therapeutic approach, it was decided to extract the ankylosed tooth 85 and subsequently apply a removable appliance, which aimed to maintain the space for tooth 45, correct the mesial inclination of tooth 46, as well as to correct crowding in the lower arch.

**Figure 2:**
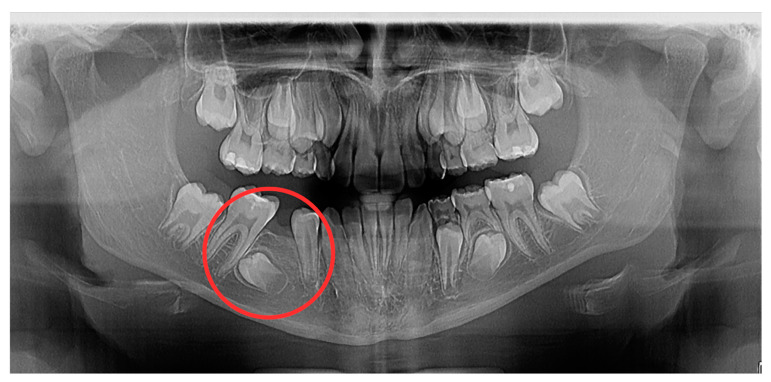
Panoramic radiograph showing the reduced space for the eruption of tooth 45 and the almost horizontal position of tooth 45.

A panoramic radiograph was taken at one year after extraction (Figure 2). The removable lower appliance corrected the incisor crowding, but the mesial inclination of tooth 46 increased. Therefore, we decided to apply a fixed space maintainer on tooth 46. The patient attended regular check-ups for approximately 20 months, but did not return for follow-up appointments for the subsequent two years.

The patient returned for a check-up at the age of 12. Clinical examination revealed facial asymmetry caused by the rightward deviation of the mandible. The anterior lower facial third was of equal height to the middle facial third. The patient presented with a straight profile, a prominent chin, and a normal nasolabial angle. The mandibular deviation to the right was functional, caused by moderate maxillary arch constriction.

The patient exhibited a half-cusp Class II dental relationship, an overjet of 2 mm, an overbite of 4 mm, a lower midline deviated to the right by 1.5 mm, moderate upper crowding, canting of the upper occlusal plane, and an infraoccluded upper right second deciduous molar (tooth 55). The fixed space maintainer was still in place.

**Figure 3:**
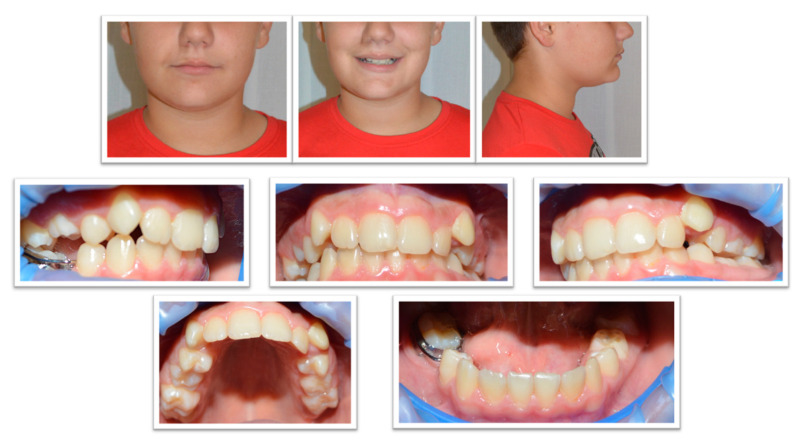
Extraoral and intraoral photographs of late mixed dentition.

**Figure 4:**
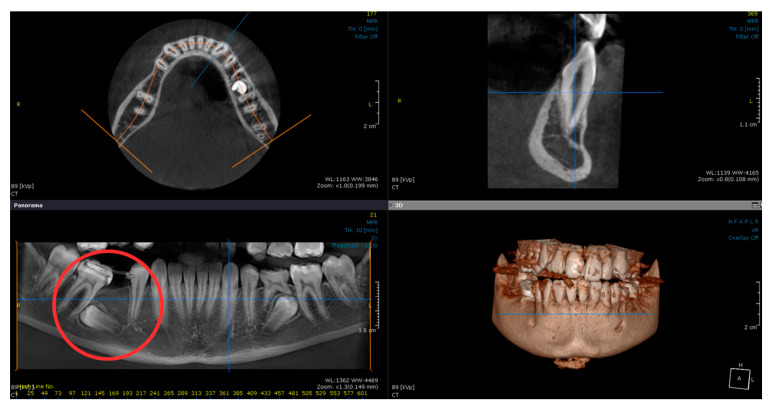
Mandibular CBCT showing the distal axial inclination of teeth 45 and 35.

Extraoral and intraoral photographs were taken at age 12, and a mandibular cone beam computed tomography (CBCT) was indicated for evaluation of the position of tooth 45 (Figure 3 and Figure 4).

### 2.2. Treatment Plan

To correct the mandibular functional shift to the right, we decided to expand the upper arch using a Haas rapid palatal expander. The expander was activated once daily, resulting in a total expansion of 6.5 mm. Due to the infraocclusion of teeth 55, 65, and 75, we decided to extract these teeth to facilitate the eruption of the second premolars.

In the second phase of treatment, we planned to apply a preadjusted edgewise bimaxillary fixed appliance in the permanent dentition. This approach aimed to align the arches, correct the occlusal cant and crowding, and create space for the eruption of tooth 45. If necessary, we also planned the surgical exposure of the impacted right lower second premolar and its traction to a temporary anchorage device (TAD).

After three months, we removed the Haas rapid palatal expander, applied a palatal bar for retention, and awaited the start of the second phase of treatment.

**Figure 5:**
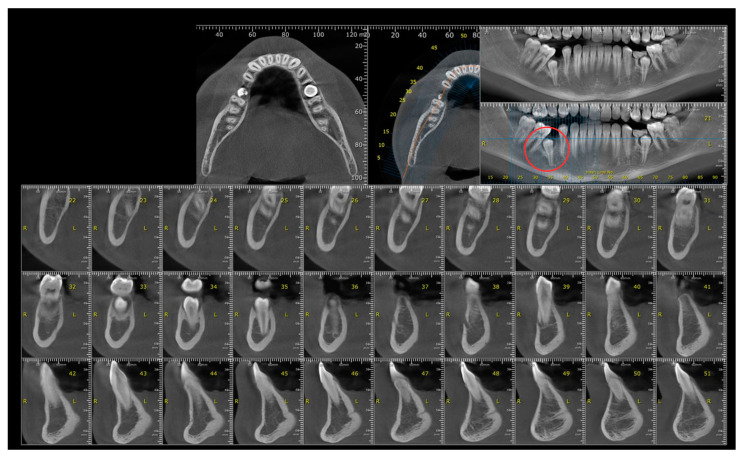
CBCT showing the proper eruption path for the second premolar.

At age 16, the patient returned to our practice and agreed to commence treatment with the preadjusted fixed edgewise appliance. We indicated a CBCT scan for the evaluation of tooth 35 (Figure 5).

### 2.3. Treatment Progress

**Figure 6:**
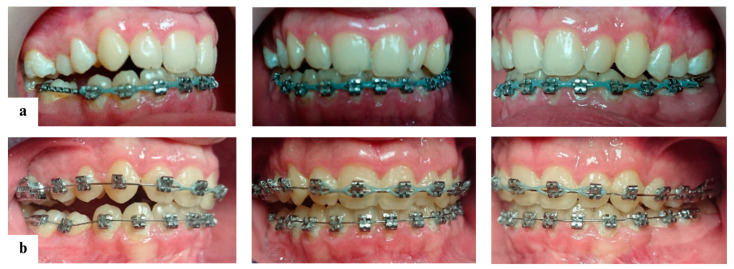
Leveling and aligning of the upper and lower arches: (**a**) 3 months of treatment with fixed appliances; and (**b**) 7 months of treatment with fixed appliances.

The treatment began with the application of preadjusted self-ligating metallic brackets, using an MBT 0.022 prescription, on both arches. Initial alignment and leveling of the upper and lower teeth were achieved using 0.014″, 0.016″, and 0.017 × 0.025″ nickel–titanium (NiTi) body heat-activated wires (Figure 6). A NiTi coil spring was used to create space for tooth 45. Once the space was sufficient, tooth 45 erupted naturally, eliminating the need for surgical exposure. A bracket was then placed on tooth 45, and it was aligned accordingly.

**Figure 7:**
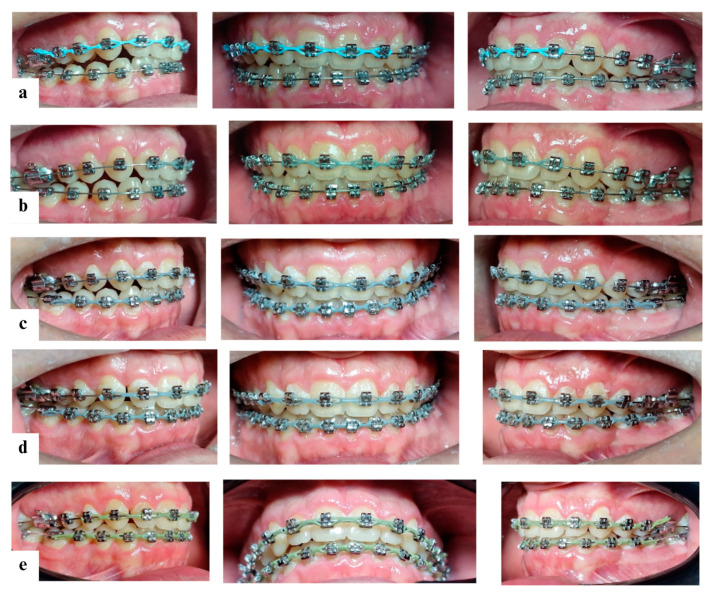
Treatment progress: (**a**) 12 months of treatment with fixed appliances; (**b**) 18 months of treatment with fixed appliances; (**c**) 21 months of treatment with fixed appliances; (**d**) 24 months of treatment with fixed appliances; and (**e**) 26 months of treatment with fixed appliances.

Final leveling and aligning were accomplished using 0.017 × 0.025″ and 0.019 × 0.025″ stainless steel (SS) archwires (Figure 7).

**Figure 8:**
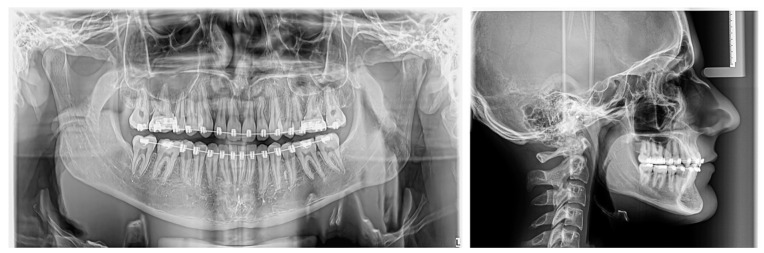
Panoramic radiograph and lateral cephalometric radiograph before debonding.

**Figure 9:**
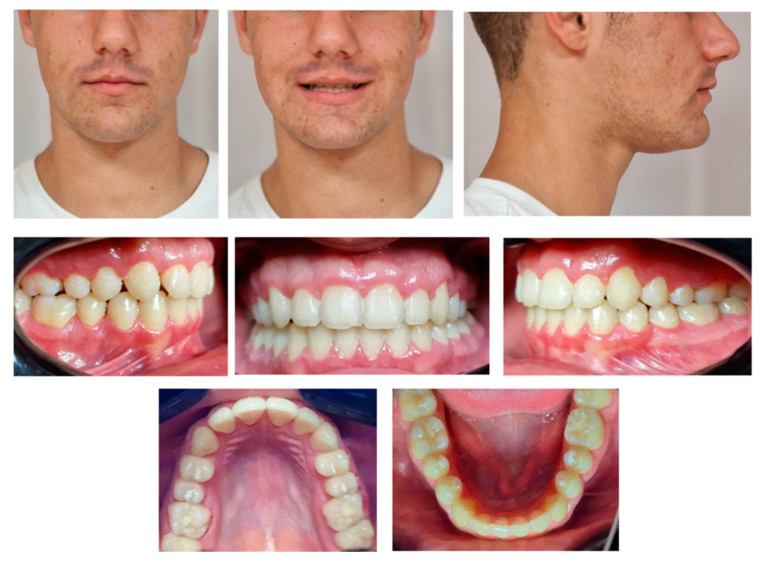
Extraoral and intraoral photographs at the end of treatment (age 18.3).

The patient wore intermaxillary elastics to improve intercuspation. Treatment with the fixed preadjusted appliance lasted 27 months (Figure 8 and Figure 9).

Based on Table 1, skeletal and dental values were within the normal range, with the exception of a slight difference noted in the U1 to SN measurement.

### 2.4. Treatment Outcome

This case report illustrates the successful conservative management of impacted mandibular second premolars in a pediatric patient, emphasizing the efficacy of early diagnosis and intervention. Through a series of carefully planned treatments, including the use of removable and fixed appliances, a Haas rapid palatal expander, and self-ligating brackets, the impacted premolars were guided to erupt naturally. Notably, the treatment obviated the need for surgical exposure. The initial use of a removable appliance corrected lower incisor crowding, setting a foundation for subsequent interventions. The application of a fixed space maintainer and subsequent fixed orthodontic appliances led to significant improvements in dental alignment and occlusal function. Progress was documented through panoramic radiographs and CBCT scans, which confirmed the favorable eruption and alignment of the impacted teeth. These results highlight the potential of conservative treatment strategies to address complex dental impactions effectively, thereby minimizing the need for more invasive procedures. Notably, the final panoramic radiograph revealed no discernible root resorption, highlighting the benefits of this carefully calibrated approach to managing complex dental impactions while minimizing the need for more invasive procedures.

## 3. Discussion

The presented case underscores the complexity and challenges associated with managing the impaction of mandibular second premolars, particularly in pediatric patients. Through a combination of early diagnosis, minimally invasive treatment approaches, and patient-specific therapeutic strategies, successful outcomes can be achieved. Early interventions in orthodontics are crucial for reducing malocclusions or their severity in the adult population [22]. Malocclusions such as posterior crossbite, Class III malocclusions, open bite, and arch length discrepancies can benefit from early orthodontic treatment and minimally invasive procedures, which reduce the complexity of these cases in the long term or even facilitate early treatment [23]. Impacted teeth or teeth with an unfavorable intraosseous eruption axis can benefit from minimally invasive treatment by simply extracting the deciduous tooth [24].

This approach was also applied in the case of the presented patient. Although the physiological age for the eruption of the lower second premolar is around 11 years [25], the extraction of the second deciduous molar, which was in infraocclusion, was indicated immediately after diagnosis when the patient was 8 years old. The early indication was warranted because infraocclusion suggests ankylosis of the temporary tooth, which, if left unaddressed, can lead to the loss of space on the dental arch, dental asymmetry, deviation of the median line, and even the re-inclusion and impaction of both the temporary tooth and the permanent successor [26]. The decision to extract infraoccluded deciduous teeth to promote the spontaneous eruption of permanent successors exemplifies a minimally invasive strategy that proved effective in this case. Similarly, Martin B. et al. (2019) presented the case of a 14-year-old patient in which the persistence of the 75 molar on the arch was visible, and the successive premolar was impacted in an almost horizontal position. In that case, the extraction was performed late, which delayed the development of the premolar. Nevertheless, after the extraction of the deciduous molar and the application of a fixed orthodontic appliance, the premolar corrected its unfavorable intraosseous position and erupted spontaneously [11].

In the case of early extraction of the deciduous molar, it is essential to preserve the space on the arch [27]. For this purpose, the presented patient was initially fitted with a removable appliance, and later with a space maintainer. The initial approach of using a lower removable appliance with an expansion screw aimed to alleviate crowding and create space for the erupting premolars. The subsequent application of a fixed space maintainer and a Haas rapid palatal expander further facilitated the correction of arch discrepancies and mandibular deviations. Otherwise, the space for the second premolar would likely have closed, making the treatment more complex and less predictable. Abu-Hussein et al. (2015) presented such a case, that of a 16-year-old patient with reduced space for the eruption of two impacted premolars. In that case, it was necessary to regain the space on the arch and perform surgical exposure and orthodontic traction of the premolar [28]. In some cases, however, regaining space is sufficient for the resumption of eruption when the tooth still has eruption potential [29].

If the treatment adopted for this patient was conservative, there are cases of impaction in which extraction remains the most suitable option [30], especially when associated with cystic formations or other complications [31]. Early intervention, as applied in the case of the presented patient, is important for the prevention of these complications. This is one of the original aspects of this case presentation. The cases identified in the specialized literature that present similar pathology refer to patients with permanent dentition, either teenagers or adults, not to children with mixed dentition.

When the time came to apply the fixed orthodontic appliance, the patient benefited from self-ligating brackets with a prescription of 0.22 MBT. These brackets integrate specialized mechanisms in the bracket and reduce or eliminate the need for external ligatures, allowing better friction control of tooth movement, and increasing the efficiency and results of the treatment [32]. Additionally, plaque accumulation is reduced and periodontal health is better compared to patients who have orthodontic appliances with conventional brackets [33].

The patient also required two CBCT examinations. One was conducted at 13 years old, focusing on the mandible, and the next at 16 years old, before the application of the fixed orthodontic appliance, covering both the maxilla and mandible. The significant advantage of this type of examination is that it allows the visualization of various dental, oral, and maxillofacial structures in multiple orthogonal images [34]. Although the use of CBCT remains limited in pediatric indications, the European Academy of Pediatric Dentistry (EAPD) has reported that CBCT may be recommended in various cases of dental and alveolar pathologies, including dental impaction [35]. It is important to note that although the effective radiation doses for CBCT are higher than those for panoramic radiographs [36], they are much lower than those required for conventional computed tomography (CT) [37]. Moreover, significant efforts are being made to optimize CBCT devices to make the radiation dose similar to, or even identical to, that required for a panoramic radiograph [34]. To minimize radiation exposure, only a lateral cephalometric radiograph was requested at the end of treatment. This allowed the identification of the final cephalometric values and the detection of growth stage CS 6 at the cervical vertebrae, indicating completed growth [38].

This case suggests that early intervention and individualized treatment plans are paramount in managing dental impactions. Clinicians should consider a combination of orthodontic and surgical techniques tailored to the patient’s specific needs and developmental stage. Regular follow-ups and the use of advanced imaging modalities, such as CBCT, are essential for monitoring treatment progress and making necessary adjustments.

This manuscript is subject to several limitations that should be acknowledged. Firstly, the article is based on a single case report, which may limit the ability to generalize treatment results to a broader population. The same therapeutic approach may yield different results in other patients. Pictures taken during the initial phases of treatment, while not of the highest quality, were useful for highlighting the infraocclusion of tooth 85. Another limitation in this case report is the absence of the initial panoramic radiograph. Unfortunately, this radiograph was provided by the parents in physical format and could not be retrieved for inclusion in this article. The potential for bias in the selection and reporting of this case, as well as the limited exploration of all possible etiological factors, further constrain the applicability of the conclusions.

Future research should include case reports that explore various approaches to managing ankylosed teeth and case reports that required force application to correctly position the premolar within the arch. This would provide a broader understanding of available therapeutic options. Additionally, studies of diverse population groups are needed to elucidate the prevalence, etiology, therapeutic management, and treatment outcomes of second premolar impaction. Such research would offer valuable insights into effective treatment strategies and enhance our understanding of this condition.

Regarding patient satisfaction, it is important to highlight that the patient expressed contentment with the outcome, and particularly appreciated the avoidance of more invasive procedures, such as extraction or surgical assisted orthodontic correction of tooth 45. Instead, the patient was happy that a minimally invasive treatment alternative was successfully employed.

## 4. Conclusions

The successful management of mandibular second premolar impaction in this pediatric patient underscores the critical importance of early diagnosis and conservative treatment approaches. The tailored use of a lower removable appliance with an expansion screw, a fixed space maintainer, a Haas rapid palatal expander, and self-ligating brackets facilitated the natural eruption of the impacted premolars without the need for surgical exposure. This case highlights the potential of minimally invasive strategies to effectively address complex dental impactions, thereby minimizing the risk of complications and the need for more invasive procedures. A key aspect of the treatment was the early extraction of the ankylosed second deciduous molar, which played a crucial role in creating space for the proper eruption of the permanent second premolar. Early extraction is essential when the premolar shows an abnormal eruption path, as it prevents further complications such as space loss, dental asymmetry, and potential impaction of both the deciduous and permanent teeth.

## Figures and Tables

**Table 1:** Lateral cephalometric measurements at the end of treatment.

Measurement	Normal Value	End of Treatment
**Skeletal**		
SNA (°)	81.9 ± 3.0	81.06
SNB (°)	78.0 ± 3.0	79.14
ANB (°)	4.0 ± 2.0	1.92
Wits (mm)	−2.0 ± 2.4	1.0
SN-GoMe (°)	36.0 ± 4.0	30.0
Gonial angle (°)	122.0 ± 6.0	124.79
**Dental**		
U1 to SN (°)	105.0 ± 5.0	97.0
L1 to GoMe (°)	95.0 ± 4.0	90.92
**Soft tissue**		
Nasolabial angle (°)	94.4 ± 10.3	114.0
Upper lip to E line (mm)	1.0 ± 2.0	10.0
Lower lip to E line (mm)	2.0 ± 2.0	9.0

SNA, angle consisting of sella, nasion, and point A; SNB, angle consisting of sella, nasion, and point B; ANB, angle consisting of point A, nasion, and point B; SN, plane consisting of sella and nasion; GoMe, plane consisting of gonion and menton; U1, upper central incisor; L1, lower central incisor; E line, line drawn from pronasale to soft tissue pogonion.

## Data Availability

The data presented in this study are available on request from the corresponding author.
